# 5WBF: a low-cost and straightforward whole blood filtration method suitable for whole-genome sequencing of *Plasmodium falciparum* clinical isolates

**DOI:** 10.1186/s12936-022-04073-1

**Published:** 2022-02-16

**Authors:** Romain Coppée, Atikatou Mama, Véronique Sarrasin, Claire Kamaliddin, Lucie Adoux, Lawrence Palazzo, Nicaise Tuikue Ndam, Franck Letourneur, Frédéric Ariey, Sandrine Houzé, Jérôme Clain

**Affiliations:** 1 Université de Paris, IRD, MERIT, 75006 Paris, France; 2 Centre National de Référence du Paludisme, AP-HP, Hôpital Bichat - Claude-Bernard, 75018 Paris, France; 3 Cumming School of Medicine, Pathology and Laboratory Medicine, The University of Calgary, Calgary, AB Canada; 4 Cochin Institute, INSERM U1016, UMR CNRS 8104, Genomic Platform, 75014 Paris, France; 5 Université de Paris, INSERM 1016, Service de Parasitologie-Mycologie Hôpital Cochin, 75014 Paris, France; 6 Present Address: Université de Paris, Infection Modelisation Antimicrobial Evolution (IAME), Inserm UMR1137, 75018 Paris, France

**Keywords:** Malaria, *Plasmodium falciparum*, Leucodepletion, Filtration, Whole-genome sequencing

## Abstract

**Background:**

Whole-genome sequencing (WGS) is becoming increasingly helpful to assist malaria control programmes. A major drawback of this approach is the large amount of human DNA compared to parasite DNA extracted from unprocessed whole blood. As red blood cells (RBCs) have a diameter of about 7–8 µm and exhibit some deformability, it was hypothesized that cheap and commercially available 5 µm filters might retain leukocytes but much less of *Plasmodium falciparum*-infected RBCs. This study aimed to test the hypothesis that such a filtration method, named 5WBF (for 5 µm Whole Blood Filtration), may provide highly enriched parasite material suitable for *P. falciparum* WGS.

**Methods:**

Whole blood was collected from five patients experiencing a *P. falciparum* malaria episode (ring-stage parasitaemia range: 0.04–5.5%) and from mock samples obtained by mixing synchronized, ring-stage cultured *P. falciparum* 3D7 parasites with uninfected human whole blood (final parasitaemia range: 0.02–1.1%). These whole blood samples (50 to 400 µL) were diluted in RPMI 1640 medium or PBS 1× buffer and filtered with a syringe connected to a 5 µm commercial filter. DNA was extracted from 5WBF-treated and unfiltered counterpart blood samples using a commercial kit. The 5WBF method was evaluated on the ratios of parasite:human DNA assessed by qPCR and by sequencing depth and percentages of coverage from WGS data (Illumina NextSeq 500). As a comparison, the popular selective whole-genome amplification (sWGA) method, which does not rely on blood filtration, was applied to the unfiltered counterpart blood samples.

**Results:**

After applying 5WBF, qPCR indicated an average of twofold loss in the amount of parasite template DNA (Pf ARN*18S* gene) and from 4096- to 65,536-fold loss of human template DNA (human *β actin* gene). WGS analyses revealed that > 95% of the  parasite nuclear and organellar genomes were all covered at ≥ 10× depth for all samples tested. In sWGA counterparts, the organellar genomes were poorly covered and from 47.7 to 82.1% of the nuclear genome was covered at ≥ 10× depth depending on parasitaemia. Sequence reads were homogeneously distributed across gene sequences for 5WBF-treated samples (n = 5460 genes; mean coverage: 91×; median coverage: 93×; 5th percentile: 70×; 95th percentile: 103×), allowing the identification of gene copy number variations such as for *gch1*. This later analysis was not possible for sWGA-treated samples, as a much more heterogeneous distribution of reads across gene sequences was observed (mean coverage: 80×; median coverage: 51×; 5th percentile: 7×; 95th percentile: 245×).

**Conclusions:**

The novel 5WBF leucodepletion method is simple to implement and based on commercially available, standardized 5 µm filters which cost from 1.0 to 1.7€ per unit depending on suppliers. 5WBF permits extensive genome-wide analysis of *P. falciparum* ring-stage isolates from minute amounts of whole blood even with parasitaemias as low as 0.02%.

**Supplementary Information:**

The online version contains supplementary material available at 10.1186/s12936-022-04073-1.

## Background

Whole-genome sequencing (WGS) has revolutionized genome-wide analyses [1]. In the context of *Plasmodium falciparum* surveillance, WGS is helpful for example to analyse the structure of parasite populations [2, 3] and to identify and track gene mutations conferring resistance to anti-malarial drugs [4, 5]. A major drawback of WGS is the large amount of human DNA compared to parasite DNA when extracted from unprocessed whole blood. Several protocols have been developed to enrich parasite DNA before WGS, either by filtering out leukocytes before DNA extraction or by selectively amplifying the parasite genome (sWGA). Current filtration procedures based on leucodepletion are however limited because they require large volumes of venous blood [6] or use either home-made cellulose-packed columns [7] or costly commercial filters [8]. Regarding sWGA-based methods [9–11], studies reported a large proportion of unmapped reads to the *P. falciparum* genome [12], the absence of coverage of the organellar genomes [9], and a wide heterogeneity in read distribution across the nuclear genome [9]. Altogether, there is a need to improve clinical sample preparation to increase data quality and exhaustiveness for *P. falciparum* genomic studies while limiting the cost of data production.

Mature red blood cells (RBCs) have a resting long diameter of about ~ 8 µm and exhibit some deformability [13]. Using a microfluidic device to examine the traversal of a RBC, the diameters of the smallest equivalent cylindrical tube, through which uninfected and parasitized RBCs could pass, were similar (2.78 and 2.79 µm, respectively) [13]. Human leukocytes are larger cells than RBCs and have diameters ranging from 9 to 21 µm depending on cell types. Hence, it was hypothesized that commercially available filters with a pore size of 5 µm might retain DNA-carrying human leukocytes but not *P. falciparum*-infected RBCs. Such a filtration could provide samples highly enriched in parasites, suitable for downstream WGS workflow. On this basis, the 5WBF method (5 µm Whole Blood Filtration), a low-cost and simple blood filtration procedure using a commercial, standardized 5 µm filter (Minisart NML® syringe, Sartorius AG, Germany), was developed. To demonstrate the usefulness of 5WBF, 400 µL of whole blood at variable parasitaemias (from 0.022 to 1.1%) was first tested from mock samples made by mixing synchronized, ring-stage cultured *P. falciparum* parasitized erythrocytes (3D7) with uninfected human whole blood. Then 5WBF was validated using 50 and 200 µL of whole blood from patients experiencing a *P. falciparum* malaria episode (ring-stage parasitaemia range: 0.04–5.5%). DNA extracts obtained after 5WBF were evaluated using the parasite:human DNA ratio assessed by qPCR and the performance of sequencing depth and percentages of coverage obtained through WGS data compared with sWGA.

## Methods

### *Plasmodium falciparum* culture and infected whole blood reconstitution

Mock whole blood samples were obtained by mixing a synchronized ring-stage *P. falciparum* culture (O-negative blood, 3D7 parasite strain) with uninfected human whole blood (final parasitaemia range: 0.022–1.1%). 3D7 parasites were cultured at 37 °C under specific atmospheric conditions (10% oxygen, 5% carbon dioxide and 85% nitrogen) in 10% human serum containing RPMI 1640 medium. One volume of pelleted culture at 10% parasitaemia was diluted in ten volumes of non-infected human whole blood. The mock sample parasitaemia was estimated to be 1.1% by Diff Quick™-stained thin blood film. The sample was then diluted 1:5 followed by another 1:10 dilution in the negative human whole blood as three independent replicates. The parasitaemias were estimated by Diff Quick™-stained, thin blood film to be 0.23% and 0.022% for the two diluted samples, respectively. These reconstituted, infected whole blood samples were hereafter called mock samples.

### Infected whole blood from *P. falciparum* malaria patients

Five fresh blood samples (collected on EDTA) from imported *P. falciparum* malaria cases diagnosed and treated at Bichat - Claude-Bernard Hospital (French Malaria Reference Centre, Paris, France) were arbitrarily selected. Diff Quick™-stained blood film examination indicated monospecific *P. falciparum* infections with ring-stage parasitaemias ranging from 0.04 to 5.5%. Additional clinical information is provided for each patient in Additional file 1: Table S1.

### 5WBF procedure

**Fig. 1:**
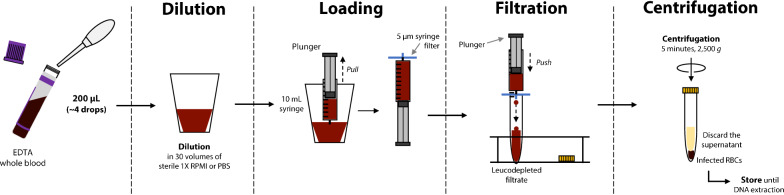
Main steps of 5WBF. From 50 to 400 µL of whole blood were diluted in RPMI 1640 medium or PBS 1× buffer in a large flask. The cartoon shows 200 µL of whole blood as an example. The diluted sample was loaded into a 10 mL syringe before the 5 μm filter was connected to the syringe. The blood was filtered by very gentle pressure (ideally, drop by drop) with the syringe plunger, until the plunger reached the bottom of the syringe to recover the maximum of infected RBCs. The filtration step itself is rapid and takes about 1 to 3 min. The filtrate was centrifuged at 2500*g* for 5 min and the supernatant was discarded. The pellet was suspended with ~ one pellet volume of RPMI 1640 or PBS 1×, transferred into a 1.5 mL tube, and stored until DNA extraction. (i) from the experiments, the filter dead volume was about 200 µL (reported as 100–150 µL by the manufacturer); (ii) even after the 2 mL optional wash with RPMI/PBS, the filter had a red colour indicating some retained RBCs or haemolysis during filtration; RBCs loss seems low although no precise quantification was done; (iii) the harder the push with the syringe plunger, the more haemolysis occurs; (iv) even with gentle push, some haemolysis can occur with some clinical samples and the filtrated pellet after centrifugation was slightly smaller, but WGS data were fine; (v) on some occasions, an air bubble could block the filter; then a slight flick at the bottom of the syringe (close to the filter) was applied; and (vi) for practical reason, a slightly different protocol was also tested in which the diluted blood sample was loaded into a 10 mL syringe after the 5 µm filter was connected to the syringe (Additional file 1: Fig. S1); similar results were obtained than with the protocol described in this Fig. 1

Prior to filtration, whole blood was diluted in either PBS 1 × buffer or RPMI 1640 medium (Fig. 1, Table 1 and Additional file 1: Fig. S1). For mock samples, 400 µL of whole blood were diluted in ten volumes of PBS 1 × buffer. For patient samples, 50 µL and 200 µL of whole blood were diluted in 30 volumes of RPMI 1640 medium, as using small sample volumes would result in large sample loss (Table 1). Each diluted blood sample was then loaded into a 10 mL syringe and filtered using a 5 µm surfactant-free cellulose acetate syringe filter (Minisart NML® syringe filter, Sartorius reference number: 17594K) connected to the syringe (Fig. 1). The sample was filtered by a very gentle push with the syringe plunger such that the filtrate flew drop by drop. Importantly, the plunger was pushed down to the bottom of the syringe. Note that even though the filtrate might pass through by gravity only, using the plunger is seemingly important to recover a maximum of infected RBCs. For mock samples only, the filter membrane was rinsed with another 2 mL of PBS 1 × buffer (Table 1). As it was noticed that skipping the rinsing step produced satisfactory WGS results, this step was not included for the second part of the study on parasite isolates from patients in order to simplify the protocol. The filtrate was then centrifuged at 2500*g* for 5 min at room temperature and the supernatant was discarded (Fig. 1). One pellet volume of RPMI 1640 (clinical samples) or PBS 1 × (mock samples) was added to the pelleted RBCs which were transferred into 1.5 mL tube and stored at 4 °C until DNA extraction within the next 24 h (Table 1). The filtration step itself is very quick (1 to 3 min), and the whole procedure takes about 20 min. Note that, for practical reason, a slightly different protocol was also tested in which the diluted blood sample was loaded into a 10 mL syringe after the 5 µm filter was connected to the syringe (Additional file 1: Fig. S1); similar results were obtained than with the protocol described in Fig. 1.

**Table 1:** Details of samples subjected to WGS

Origin	Sample name	Enrichment method	% para	Blood volume (µL)	Blood:buffer dilution	Washing filter membrane^a^	Volume of DNA elution buffer (µL)^b^
Mock (3D7)	M1	None	1.10	400	n.a	n.a	200
	M1_WGA_	sWGA	1.10	400	n.a	n.a	200
	M1-1_5F_	5WBF	1.10	400	1:10, PBS 1×	2 mL PBS 1×	200
	M1-2_5F_	5WBF	1.10	400	1:10, PBS 1×	2 mL PBS 1×	200
	M1-3_5F_	5WBF	1.10	400	1:10, PBS 1×	2 mL PBS 1×	200
	M2_WGA_	sWGA	0.23	400	n.a	n.a	200
	M2-1_5F_	5WBF	0.23	400	1:10, PBS 1×	2 mL PBS 1×	200
	M2-2_5F_	5WBF	0.23	400	1:10, PBS 1×	2 mL PBS 1×	200
	M2-3_5F_	5WBF	0.23	400	1:10, PBS 1×	2 mL PBS 1×	200
	M3_WGA_	sWGA	0.022	400	n.a	n.a	200
	M3-1_5F_	5WBF	0.022	400	1:10, PBS 1×	2 mL PBS 1×	200
	M3-2_5F_	5WBF	0.022	400	1:10, PBS 1×	2 mL PBS 1×	200
	M3-3_5F_	5WBF	0.022	400	1:10, PBS 1×	2 mL PBS 1×	200
Patients	P1_5F-50_	5WBF	0.04	50	1:30, RPMI 1640	n.d	200
	P1_5F-200_	5WBF	0.04	200	1:30, RPMI 1640	n.d	200
	P2_5F-50_	5WBF	0.08	50	1:30, RPMI 1640	n.d	200
	P2_5F-200_	5WBF	0.08	200	1:30, RPMI 1640	n.d	200
	P3_5F-50_	5WBF	0.25	50	1:30, RPMI 1640	n.d	200
	P3_5F-200_	5WBF	0.25	200	1:30, RPMI 1640	n.d	200
	P4_5F-50_	5WBF	0.40	50	1:30, RPMI 1640	n.d	200
	P4_5F-200_	5WBF	0.40	200	1:30, RPMI 1640	n.d	200
	P5_5F-50_	5WBF	5.50	50	1:30, RPMI 1640	n.d	200
	P5_5F-200_	5WBF	5.50	200	1:30, RPMI 1640	n.d	200

% para: parasitaemia in percentage; n.a.: not applicable; n.d.: not done

^a^Rinsing the filter with 2 mL of buffer after blood filtration is optional: as skipping the rinsing step produced satisfactory WGS results, this step was not performed for the second part of this study on parasite isolates from patients in order to simplify the protocol

^b^Elution buffer refers to DNA extraction from the unfiltered/filtered blood samples

As a negative control for filtration, whole blood (400 µL) for one mock sample (parasitaemia of 1.1%) was subjected to the same pipeline similarly to other mock samples, except that no filter was connected at the bottom of the syringe (Table 1). This control mock sample was latter called M1.

### DNA extraction

DNA extraction was carried out on unfiltered and 5WBF-treated samples using the MagPurix® Blood DNA Extraction Kit 200 (Biosynex, France), then eluted using the elution buffer according to the manufacturer’s recommendations (Table 1). DNA was quantified using the Qubit® dsDNA high sensitivity kit (Thermo Fisher Scientific) according to the manufacturer’s recommendations.

### sWGA procedure

The sWGA method was performed on genomic DNA from unfiltered samples according to published protocols [9]. The sWGA reaction was performed in 0.2 mL PCR-tubes, containing 10 ng of template genomic DNA, 1 × BSA (New England Biolabs), 1 mM dNTPs (New England Biolabs), 2.5 µM of each amplification primer (Additional file 1: Table S2), 1 × Phi29 reaction buffer (New England Biolabs), 30 units of Phi29 polymerase (New England Biolabs), and molecular biology grade water to reach a final reaction volume of 50 µL. The reaction was carried out on a thermocycler with the following step-down program: 5 min at 35 °C, 10 min at 34 °C, 15 min at 33 °C, 20 min at 32 °C, 30 min at 31 °C, 16 h at 30 °C, then heating for 15 min at 65 °C to inactivate the Phi29 polymerase before cooling to 4 °C. Amplified products were quantified using the Qubit® dsDNA high sensitivity kit (Thermo Fisher Scientific) to determine whether there was at least 500 ng of product for sequencing. Amplified products were cleaned using Agencourt Ampure XP beads (Beckman Coulter) as follows: 1.8 volumes of beads were added to 1 volume of amplified products, briefly mixed, and then incubated for 5 min at room temperature. A magnetic rack was used to capture the DNA binding beads. The DNA binding beads were then washed twice using 200 µL of 80% ethanol and eluted with 60 µL of EB buffer.

### Quantitative PCR

The copy number of specific *P. falciparum* and human genes within the genomic DNA from patient blood samples was estimated by qPCR with *Plasmodium* Typage kit (Bio-Evolution, France). Briefly, as recommended by the manufacturer, 5 µL of DNA extract was mixed with 15 µL of Master Mix containing specific primers and probes targeting *P. vivax* and *P. falciparum* ARN*18S* and human *β actin* genes. The reaction was carried out on a thermocycler (ViiA 7, Applied Biosystems) with the following program: 30 s at 95 °C; 40 cycles: 15 s at 95 °C followed by 45 s at 60 °C; then 1 s at 37 °C. Positive and negative controls were included in each run.

### Whole-genome sequencing

250 ng of DNA were used for mechanical DNA shearing that was performed in a total volume of 52 µL with the Covaris S220 through microTube-50 AFA Fiber Screw-Cap (Covaris®) using a setting of 30% duty factor, 100 W peak incidence power, and 1000 cycles per burst for 150 s. Note that the concentrations of genomic DNA from 5WBF-treated blood samples were often very low or even below the Qubit® detection threshold (Additional file 1: Table S3). Then, 52 µL of undiluted genomic DNA were used for mechanical DNA shearing. Genomic DNA libraries were constructed for high throughput sequencing using the KAPA HyperPrep Library Preparation Kit (Kapa Biosystems, Woburn, MA). DNA libraries were checked for quality and quantity using Qubit® for concentration and BioAnalyser 2100 Agilent for fragment size. Libraries were sequenced at 150 bp paired-end using an Illumina NextSeq 500 instrument at the GENOM’IC platform from Institut Cochin (Paris, France).

### Sequencing output analysis

Sequence data obtained from each sample was subjected to standard Illumina QC procedures. Each sample was analysed independently by mapping sequence reads to the *P. falciparum* 3D7 reference genome v.39 using the Burrows-Wheeler Aligner (BWA) software package [14]. Samtools (http://samtools.sourceforge.net/) was used to generate coverage statistics and depth estimates from the BWA mapping output. Qualimap v2.2.1 was also used to perform an analysis based on specific features derived from the alignment, including coverage, GC content and mapping quality [15]. A home-made python script was developed to calculate the percentage of each *P. falciparum* gene covered at ≥ 10 × depth and the per-gene mean coverage depth (https://github.com/Rcoppee/Scan_gene_coverage). The script required a reference genome file in fasta format, an annotation gff file indicating the location of each exon, intron and corresponding genes, and a per-base coverage depth file generated with Bedtools *genomecov* function using default parameters [16]. This per-base coverage depth file was also used to plot the average read depth within 1-kb windows across the 14 *P. falciparum* chromosomes using the Circos software [17]. Finally, per-gene copy number was assessed using PlasmoCNVScan, a custom read depth strategy specifically made for *Plasmodium* species [18].

### Ethical considerations

Samples received at the French Malaria Reference Centre (Paris, France) were registered and declared for research purposes as a biobank for both the Assistance Publique des Hôpitaux de Paris and Santé Publique France. The uninfected blood sample was obtained from a patient having a negative malaria test. No institutional review board approval was required according to French legislation (article L. 1111-7 du Code de la Santé Publique, article L. 1211-2 du Code de Santé Publique, articles 39 et suivants de la loi 78-17 du 6 janvier 1978 modifiée en 2004 relative à l’informatique, aux fichiers, et aux libertés).

## Results

### Application of 5WBF to mock samples

**Fig. 2:**
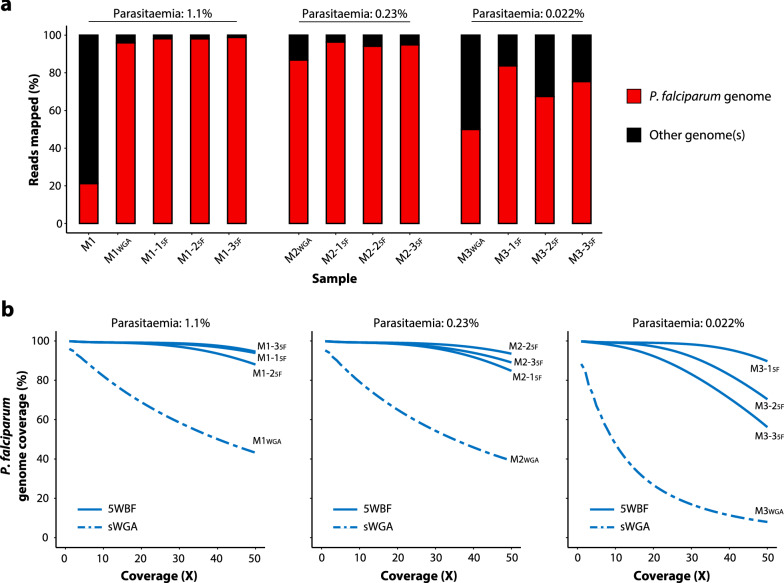
Proportions of mapped reads and *P. falciparum* genome coverage from mock whole blood samples. **a** Proportion of reads mapping to the *P. falciparum* nuclear and organellar genomes. Red and black colours indicate the proportion of reads mapping and not mapping to the *P. falciparum* genomes respectively. **b** Genome fraction coverage from 1× to 50× depth. Data from sWGA- and 5WBF-treated samples are indicated in dashed and solid lines, respectively. Three independent 5WBF blood filtration replicates were made for each parasitaemia

5WBF was first applied to 400 µL of mock blood samples (3D7 culture diluted in uninfected whole blood) with parasitaemias of 1.1, 0.23 and 0.022% (each in triplicate). For the unfiltered control mock sample (sample M1; 1.1% parasitaemia), 21.2% of the reads mapped to the *P. falciparum* genome (Fig. 2a) with a mean coverage of 5.6× depth (Table 2). For the 5WBF-treated mock samples (n = 9), an average of 89.6% (standard deviation: 11.4) of the reads mapped to the *P. falciparum* genome (Fig. 2a). The proportion of *P. falciparum*-mapped reads decreased with parasitaemia (Fig. 2a and Table 2). Regardless, at least 98.6% of the *P. falciparum* genome was covered at ≥ 10× depth whatever the parasitaemia (Fig. 2b). Reads also covered both the parasite’s mitochondrial and apicoplast genomes, for which mean coverages were systematically higher than 1000× and 59.6× depths respectively. For comparison, one unfiltered mock sample of each parasitaemia was processed by the sWGA procedure. Lower proportions of reads mapped to the *P. falciparum* genome when from sWGA-treated compared to 5WBF-treated samples (Fig. 2a and Table 2). The difference was modest at 1.1% parasitaemia but it increased as parasitaemias dropped to 0.23 and 0.022%. From 47.7 to 82.1% of the nuclear genome was covered at ≥ 10× depth depending on parasitaemia (Fig. 2b). Also, reads from sWGA poorly covered the parasite mitochondrial and apicoplast genomes, systematically below a mean coverage of 10× depth (Table 2). In summary almost all the bases of the different genomes of *P. falciparum* were analysable at ≥ 10 × depth using 400 µL of 5WBF-treated whole blood.

**Table 2:** WGS statistics of *P. falciparum* DNA extracted from mock samples subjected to either no treatment or sWGA or 5WBF

Sample name	% para	Enrichment method	Total reads	*P. falciparum*-mapped reads (×)	Mean genome coverage (×)	Mean coverage of mt genome (×)	Mean coverage of api genome (×)
M1	1.1	None	14,777,245	3,135,321 (21.22)	5.60	45.39	11.76
M1_WGA_	1.1	sWGA	16,002,711	15,347,302 (95.9)	74.41	5.20	8.44
M1-1_5F_	1.1	5WBF	19,932,109	19,558,272 (98.12)	104.86	2102.17	216.15
M1-2_5F_	1.1	5WBF	16,923,729	16,591,158 (98.03)	89.59	1701.08	183.29
M1-3_5F_	1.1	5WBF	17,230,853	17,018,367 (98.77)	90.04	1335.70	150.66
M2_WGA_	0.23	sWGA	16,266,710	14,112,713 (86.76)	69.16	1.59	3.57
M2-1_5F_	0.23	5WBF	14,905,078	14,350,236 (96.28)	79.71	1468.76	120.40
M2-2_5F_	0.23	5WBF	19,310,196	18,169,487 (94.09)	99.82	1782.77	161.75
M2-3_5F_	0.23	5WBF	17,672,315	16,766,350 (94.87)	91.15	1933.32	129.47
M3_WGA_	0.022	sWGA	9,840,798	4,915,197 (49.95)	18.63	0.31	2.34
M3-1_5F_	0.022	5WBF	17,283,136	14,467,900 (83.71)	76.72	1209.77	115.57
M3-2_5F_	0.022	5WBF	17,759,091	11,975,821 (67.43)	62.32	1528.46	98.56
M3-3_5F_	0.022	5WBF	13,052,530	9,839,100 (75.38)	52.17	1026.41	59.63

Reads were mapped to the *P. falciparum* 3D7 reference genome v.39

% para: parasitaemia in percentage; mt: mitochondrial; api: apicoplast

Then two gene-level metrics were explored: the percentage of each *P. falciparum* gene covered at ≥ 10× depth and corresponding mean coverage. For these analyses, one 5WBF-treated sample (M2-1_5F_) and one sWGA-treated sample (M2_WGA_) that presented a similar number of reads mapping to the *P. falciparum* genome were used (~ 14 million reads; Table 2).

**Fig. 3:**
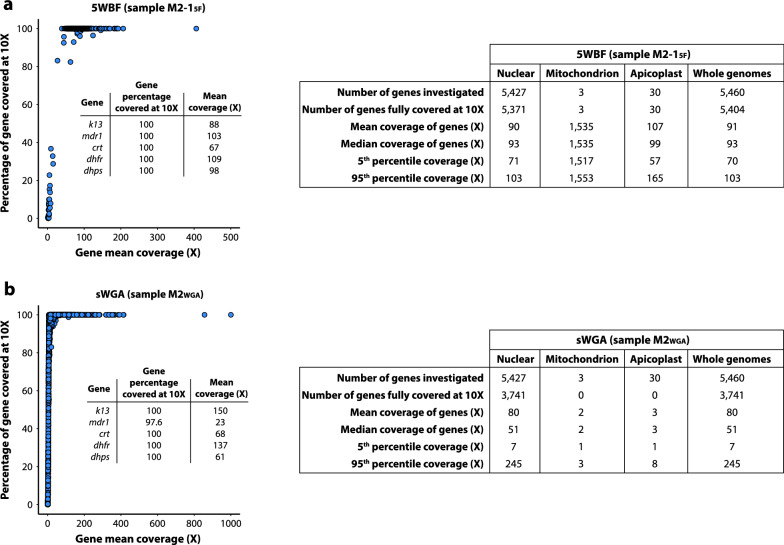
Comparison of gene coverage depth between M2_WGA_ and M2-1_5F_ mock samples. **a** Coverage depth and gene percentage covered at ≥ 10× depth of all genes for M2-1_5F_. Each blue point corresponds to a gene. Mitochondrial genes were discarded for ease of representation. The *insert* table indicates the mean coverage and the percentage of genes covered at ≥ 10× depth for five drug resistance genes. Descriptive statistics on the right table included the total number of *P. falciparum* (3D7) genes, the number of genes fully covered at ≥ 10× depth, the mean and median coverage of all genes, and the 5th and 95th percentiles of coverage depth. Genes were partitioned as of either nuclear, mitochondrial, or apicoplast origins. **b** Coverage depth of all genes for M2_WGA_. Description of the plot and the tables are the same as in **a**

First, for the 5WBF-treated sample (M2-1_5F_), 99.0% (5404/5460) of nuclear genes and all organellar genes were fully covered at ≥ 10× depth, including important drug resistance genes such as *k13*, *mdr1*, *crt*, *dhfr* and *dhps* (Fig. 3a). The few uncovered genes were mostly *rifin* and *var*. Using sWGA, 68.5% (3741/5460) of *P. falciparum* genes were fully covered at ≥ 10× depth. None of the mitochondrial and apicoplast genes were covered at ≥ 10× depth, and the drug resistance gene *mdr1* was not fully covered at this threshold (Fig. 3b).

**Fig. 4:**
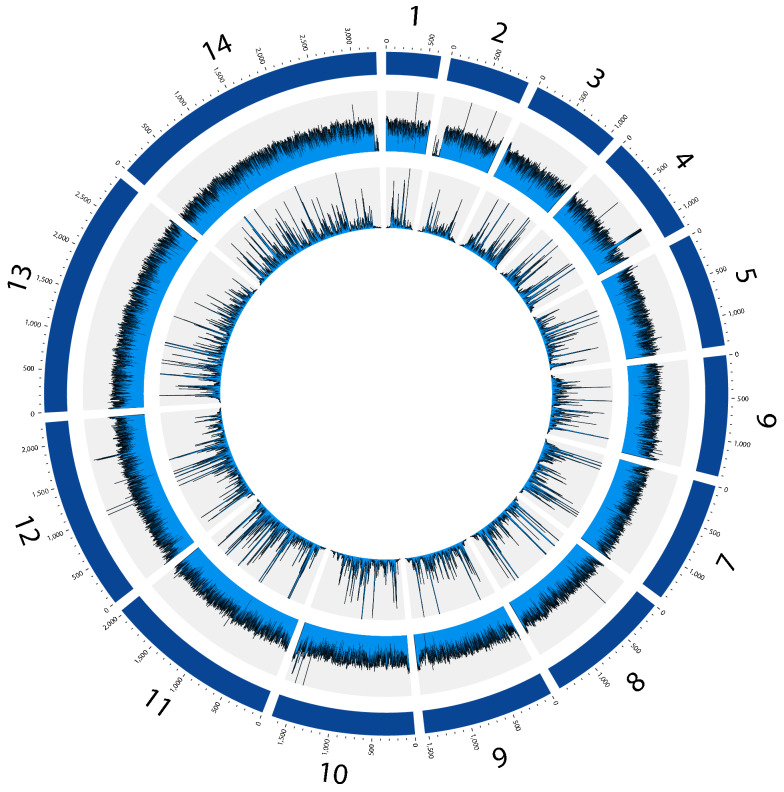
Distribution of the reads along the 14 chromosomes of the *P. falciparum* nuclear genome for M2-1_5F_ and M2_WGA_ mock samples. The three rings represent, from outermost to innermost, the 14 chromosomes of the *P. falciparum* nuclear genome (illustrated to scale in kb), and the average read depth within 1-kb windows for M2-1_5F_ and for M2_WGA_, respectively. For ease of representation, the maximum depth for M2-1_5F_ and M2_WGA_ was fixed at 200× and 500×, respectively

Second, the coverage per gene varied little with the 5WBF sample (mean coverage: 91×; median: 93×; 5th percentile: 70×; 95th percentile: 103×) compared with the sWGA sample (mean coverage: 80×; median: 51×; 5th percentile: 7×; 95th percentile: 245×) (Fig. 3a and b). The coverage depth was also measured at each base across the 14 chromosomes of the *P. falciparum* nuclear genome. Reads mapped homogeneously across the genome with 5WBF, while they mapped much more heterogeneously in sWGA (Fig. 4). Consequently, 5WBF is likely compatible with analyses based on read distribution such as identifying per-gene copy number.

### Application of 5WBF to *P. falciparum* clinical isolates

The 5WBF procedure was then tested on *P. falciparum* clinical samples with parasitaemias ranging from 0.04 to 5.5% (Table 3). For that, 50 µL (i.e. one drop) and 200 µL of whole blood from patients were used to match blood volumes routinely collected in a clinical context.

**Table 3:** 5WBF-based WGS statistics from five *P. falciparum* clinical isolates

Patient	% para	Sample name	Volume of blood filtered (µL)	Total reads	*P. falciparum*-mapped reads (%)	Mean genome coverage (×)	Mean coverage of mt genome (×)	Mean coverage of api genome (×)
P1	0.04	P1_5F-50_	50	12,179,133	9,487,373 (77.90)	49.97	664.63	38.04
		P1_5F-200_	200	11,913,069	11,363,989 (95.39)	60.43	910.04	45.12
P2	0.08	P2_5F-50_	50	11,305,296	7,949,064 (70.31)	40.96	534.58	53.71
		P2_5F-200_	200	10,668,907	9,273,820 (86.92)	49.09	807.60	61.40
P3	0.25	P3_5F-50_	50	11,727,334	10,693,769 (91.19)	59.79	490.97	48.93
		P3_5F-200_	200	15,203,526	14,512,154 (95.45)	77.68	628.79	80.60
P4	0.40	P4_5F-50_	50	12,500,393	8,627,931 (69.02)	44.39	449.46	79.74
		P4_5F-200_	200	13,886,282	12,952,939 (93.28)	68.77	779.27	106.35
P5	5.50	P5_5F-50_	50	22,830,831	22,459,337 (98.37)	117.53	1219.30	240.98
		P5_5F-200_	200	28,206,251	27,924,816 (99.00)	147.98	1483.73	357.97

% para: parasitaemia in percentage; mt: mitochondrial; api: apicoplast

First, parasite and human DNA amount were assessed by qPCR, expressed in Ct (cycle threshold), before and after 5WBF (Additional file 1: Table S3). Slightly higher Ct values were observed after 5WBF for the parasite qPCR assay (Ct_5WBF_ – Ct_unfiltered_: mean = 1.1, min = -1, max = 3; n = 5 samples). In contrast, a dramatic increase in Ct values was observed after 5WBF for the human qPCR assay (Ct_5WBF_ – Ct_unfiltered_: mean = 14, min = 10, max = 16; n = 5 samples). Accordingly, the amount of total genomic DNA quantified by Qubit was dramatically lower in the 5WBF-treated samples compared to their unfiltered counterparts (Additional file 1: Table S3).

**Fig. 5:**
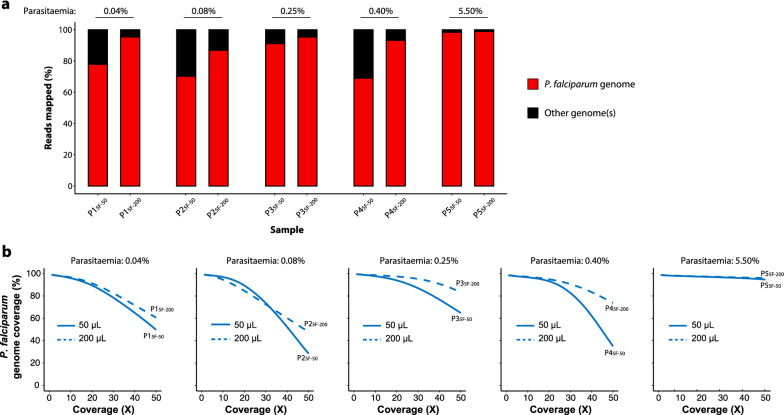
Proportions of mapped reads and *P. falciparum* genome coverage from clinical samples. **a** Proportion of reads mapping to the *P. falciparum* nuclear and organellar genomes. Red and black colours respectively indicate the proportion of reads mapping and not mapping to the *P. falciparum* genomes. **b** Genome fraction coverage from 1× to 50× depth. Data from 50 µL and 200 µL of filtered whole blood are indicated in solid and dashed lines, respectively

With WGS data, an average of 81.4% (standard deviation: 13.0) and 94.0% (standard deviation: 4.5) of the reads mapped to the *P. falciparum* genome when 50 µL and 200 µL of whole blood were filtered, respectively (Fig. 5a and Table 3). The mean genome coverage, including organellar genomes, was systematically higher for 200 µL than 50 µL of whole blood (Table 3). However, at the 10× depth threshold, the *P. falciparum* genome coverage was similar whether using 50 µL or 200 µL of whole blood (average: 97%; standard deviation: 1.2; Fig. 5b).

**Fig. 6:**
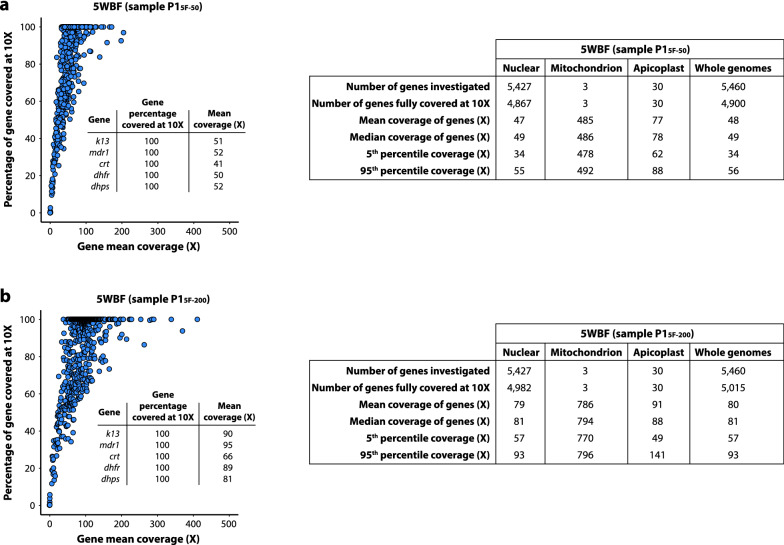
Comparison of gene coverage depth between P1_5F-50_ and P1_5F-200_ 5WBF-treated clinical samples. **a** Coverage depth and gene percentage covered at ≥ 10× depth of all genes for P1_5F-50_. Each blue point corresponds to a gene. Mitochondrial genes were discarded for ease of representation. The *insert* table indicates the mean coverage and the percentage of gene covered at ≥ 10× depth of five drug resistance genes. Descriptive statistics on the right table included the total number of *P. falciparum* (3D7) genes, the number of genes fully covered at ≥ 10× depth, the mean and median coverage of all genes, and the 5th and 95th percentiles of coverage depth. Genes were partitioned as of either nuclear, mitochondrial, or apicoplast origins. **b** Coverage depth of all genes for P1_5F-200_. Description of the plot and the tables are the same as in **a**

**Fig. 7:**
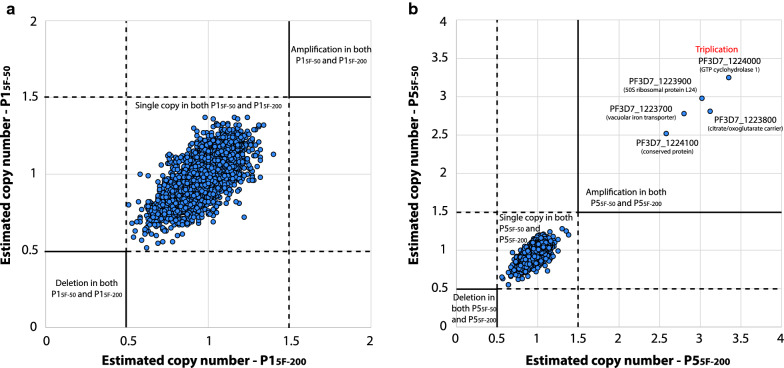
Estimation of per-gene copy number for clinical samples using WGS data and the PlasmoCNVScan program. Per-gene copy number was shown for the P1_5F-50_ and P1_5F-200_ samples (**a**), and for the P5_5F-50_ and P5_5F-200_ samples (**b**). Each point corresponds to a gene. A value < 0.5 suggests a gene deletion, while a value > 1.5 suggests a gene amplification. Values between 0.5 and 1.5 suggests a single copy gene. A positive correlation was observed for gene copy numbers estimated using 50 µL and 200 µL of blood for a same isolate (Spearman’s rank correlation: *p* < 0.001 and r = 0.72 for the P1_5F-50_ and P1_5F-200_ paired samples; *p* < 0.001 and r = 0.84 for the P5_5F-50_ and P5_5F-200_ paired samples)

Then the same two gene-level metrics were explored as previously done for mock samples. For these analyses, the 50 µL and 200 µL samples from the patient P1 were selected (parasitaemia of 0.04%; P1_5F-50_ and P1_5F-200_; Table 3) because they presented a roughly similar number of reads mapping to the *P. falciparum* genome. 89.7% (4900/5460) and 91.8% (5015/5460) of *P. falciparum* genes were fully covered at ≥ 10 × depth with P1_5F-50_ and P1_5F-200,_ respectively, including the major drug resistance genes and all organellar genes (Fig. 6). The coverage (×) per gene metrics (mean, median, and 5th and 95th percentiles) were roughly twice larger for P1_5F-200_ compared to P1_5F-50_, except for apicoplast genes (Fig. 6). The per-gene copy number was then compared for P1_5F-50_ and P1_5F-200_. Beforehand, all the variant surface antigens gene families (*var*, *stevor*, *rifin*, *phist* and *Plasmodium exported protein-*encoding genes) were removed to avoid any bias in the analysis (4816 remaining genes). Similar profiles were obtained for P1_5F-50_ and P1_5F-200_ and no gene amplification was detected for P1 isolate (Spearman’s rank correlation: *p* < 0.001, r = 0.72; Fig. 7a). Among the other tested samples, P5_5F-50_ and P5_5F-200_ each harbored three copies of the *GTP cyclohydrolase 1* gene (*gch1*; PF3D7_1224000) and of the four genes neighbouring *gch1* (PF3D7_1223700, PF3D7_1223800, PF3D7_1223900 and PF3D7_1224100; Fig. 7b). The total amplicon size was 10.5 kb and resembled to the one detected in Thai isolates [19]. Altogether, 50 µL of whole blood at 0.04% parasitaemia treated by 5WBF permitted to explore per-gene copy number in a clinical context.

## Discussion

The ability to produce high-quality sequencing data from *P. falciparum* clinical samples has valuable implications for public health. The sWGA method has massively facilitated the generation of WGS from clinical whole blood samples stored on dried blood spot (DBS). However, sWGA-based methods present several drawbacks. Current primers used to selectively amplify the *P. falciparum* genome lead to the nearly complete loss of the mitochondrial and apicoplast genomes [9]. Furthermore, a large proportion of reads often do not map to the *P. falciparum* genome, suggesting that some contaminant human DNA remains after the sWGA step [12] and which increases the sequencing cost. Finally, reads mapping to the reference genome are not homogeneously distributed across the genome, precluding any investigation based on read distribution such as the measure of per-gene copy number [18].

Here, the usefulness of 5WBF as a new leucodepletion protocol based on commercial 5 µm filters was shown. Other filtration approaches were already successfully developed but also have their own drawbacks—whether in terms of costs, blood volumes, or standardization [7, 8]. In this work, 5WBF was used for WGS purposes, but this strategy may likely be useful for other sequencing applications, such as RNAseq, which often suffers from contaminant human DNA when applied to *P. falciparum* isolates and thus requiring additional costs.

Sequencing data obtained with whole blood samples treated by 5WBF revealed that all the three *P. falciparum* genomes (nuclear, mitochondrial and apicoplast) were covered with high coverage depth. Almost all of the *P. falciparum* genes were fully covered at ≥ 10 × depth, except the highly variable *var* and *rifin* gene families. Capturing the organellar genomes is especially important since they carry drug resistance genes, such as *cytb* or *rps4* [20–22], or can inform on the geographic origin and evolution of the parasites [23]. Finally, the homogeneous distribution of reads across the genome makes it possible to detect gene copy number variations, some of which are genotypic markers of anti-malarial drug resistance [24–26].

The 5WBF procedure was extensively tested here using the 5 µm Minisart NML® syringe filter from the manufacturer Sartorius. Other commercially available 5 µm might also be suitable and would need validation experiments. Financially, the cost of the 5 µm Minisart NML® syringe filter varies from 1.0 to 1.7€ per unit depending on suppliers. This seems about 10 times cheaper than the Plasmodipur filter (Europroxima, Arnhem, The Netherlands, Cat. 8011Filter25U) [6, 8]. Similarly, sWGA-based methods are more expensive than 5WBF since Phi29 DNA polymerase, primer sets and subsequent purification with Agencourt Ampure XP beads increase the cost to approximately 6–8€ per sample [9–11]. The leucodepletion-based method through CF11 cellulose column likely has a roughly similar implementation cost than 5WBF [7]. However, these are homemade columns and thus requires an extended preparation time, and likely lack the standardized and certified quality of commercially available filters. Altogether, the 5WBF procedure provides remarkable add-ons: simplicity and speed of the filtration procedure, standardized and ready-to-use sterile filters, low cost per sample, and high quality of WGS data. Also, 5WBF-treated blood samples could probably be easily stored after centrifugation as DBS on filter papers, in the exact same way as was previously done with Plasmodipur filtration [8]. This possibility remains to be tested.

The 5WBF procedure has some limitations. First, as for any filtration procedure, it introduces practical constraints related to the centrifugation of the resulting filtrate to pellet RBCs. If no power is available on preparation site, the 5WBF-filtered RBCs could then be left to precipitate for approximately 3 h as previously done [8]. Second, it was successful mainly for blood samples infected with *P. falciparum* parasites at the ring-stage, which correspond to the vast majority of *P. falciparum* clinical isolates. In 5WBF experiments with asynchronous in vitro 3D7 parasite culture, erythrocytes infected with more mature *P. falciparum* stages like mature trophozoites and schizonts were not consistently recovered in the 5WBF filtrate. Of note, the filtrated ring-stage 3D7 parasites were viable and could mature and replicate in culture. Few *Plasmodium ovale, Plasmodium malariae* and *Plasmodium vivax* clinical samples were also tested and a large parasite DNA loss was obtained in some samples after 5WBF. Therefore, at this stage, it is not recommended to use the 5WBF method to recover erythrocytes infected by non-*falciparum* species, being co-infections or not, nor by more mature *P. falciparum* stages like mature trophozoites and schizonts.

## Conclusion

In summary, 5WBF is a simple and cheap filtration procedure that depletes leukocytes from human blood. 5WBF treatment of minute amounts of clinical blood samples permits extensive genome-wide analysis of *P. falciparum*, including the coverage of organellar genomes and the detection of gene copy number variations.

## Supplementary Information

**Additional file 1: Figure S1.** Alternative 5WBF protocol. **Table S1.** Clinical information of the patients included in the study. **Table S2.** sWGA primers for *P. falciparum*. **Table S3.** Content in *P. falciparum* and total DNA before and after 5WBF measured by qPCR and Qubit.

## Data Availability

The script used to calculate the percentage of each *P. falciparum* gene covered at ≥ 10 × depth and the per-gene mean coverage depth was deposited on github: https://github.com/Rcoppee/Scan_gene_coverage. The datasets analysed in the study are available from the corresponding authors on request.

## Abbreviations

5WBF: 5 µM Whole Blood Filtration
Ct: Cycle threshold
DBS: Dried blood spots
RBCs: Red blood cells
sWGA: Selective whole-genome amplification
WGS: Whole-genome sequencing
