# Supplementary material for: 5WBF: a low-cost and straightforward whole blood filtration method suitable for whole-genome sequencing of Plasmodium falciparum clinical isolates
Source: Malar J. 2022 Feb 16;21:51. doi: 10.1186/s12936-022-04073-1 (PMC8848818; doi:10.1186/s12936-022-04073-1)
Supplement: Supplementary file 1 — Additional file 1: Figure S1. Alternative 5WBF protocol. Table S1. Clinical information of the patients included in the study. Table S2. sWGA primers for P. falciparum. Table S3. Content in P. falciparum and total DNA before and after 5WBF measured by qPCR and Qubit. [file 12936_2022_4073_MOESM1_ESM.docx]

**Additional file 1 : Supplementary Figures and Tables**

This supplemental file has been provided by the authors to give readers additional information about their work.

Supplement to: **5WBF: A low-cost and straightforward whole blood filtration method suitable for whole-genome sequencing of *Plasmodium falciparum* clinical isolates**

By Romain Coppée, Atikatou Mama, Véronique Sarrasin, Claire Kamaliddin, Lucie Adoux, Lawrence Palazzo, Nicaise Tuikue Ndam, Franck Letourneur, Frédéric Ariey, Sandrine Houzé, Jérôme Clain

**Figure S1 ……………………….……………………………………….……………………………. 2**

**Table S1 …………………………………………………………………………………….…..…….. 3**

**Table S2 …………………………………………………………………………………….…..…….. 3**

**Table S3 …………………………………………………………………………………….…..…….. 3**


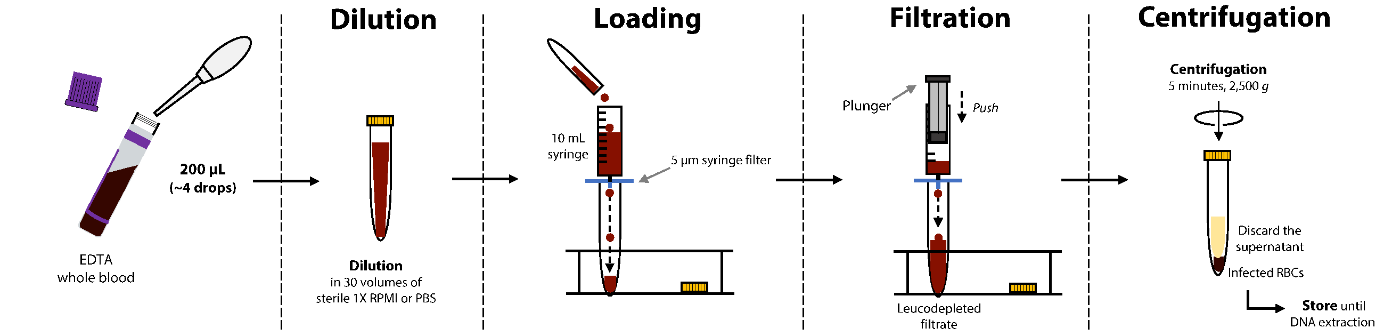


**Figure S1 – Alternative protocol of 5WBF tested in our lab**. 50 to 400 μL of whole blood were diluted in RPMI 1640 medium or PBS 1X buffer in a 15 mL tube. The cartoon shows 200 μL of whole blood as an example. The diluted sample was loaded onto a 10 mL syringe after the 5 μm filter was connected to the syringe. The blood was filtered by very gentle pressure (ideally, drop by drop) with the syringe plunger, until the plunger reached the bottom of the syringe to recover the maximum of infected RBCs. The filtration step itself is rapid and takes about 1 to 3 minutes. The filtrate was centrifuged at 2,500 *g* for 5 minutes and the supernatant was discarded. The pellet was suspended with ~ one pellet volume of RPMI 1640 or PBS 1X, transferred into a 1.5 mL tube, and stored until DNA extraction. **Important notes**:

*i*) from our experiments, we estimated that the filter dead volume was about 200 µL (reported as 100-150 µL by the manufacturer).

*ii*) after filtration the filter had a red color indicating some retained RBCs or hemolysis during filtration; RBCs loss seems low although it not quantified.

*iii*) the harder the push with the syringe plunger, the more hemolysis occurs.

*iv*) even with gentle push, some hemolysis can occur with some clinical samples and the filtrated pellet after centrifugation was slightly smaller, but NGS data were fine.

*v)* on some occasions, an air bubble could block the filter; then a slight flick at the bottom of the syringe (close to the filter) was applied.

**Table S1 – Clinical information of the patients included in the study.**

| **Patient** | **Sex** | **Age** | **Infection country** | **Prophylaxis** | **% para^a^** | **Volume of blood filtered (µL)** | **Sample name** |
| --- | --- | --- | --- | --- | --- | --- | --- |
| 1 | M | 20 | Ivory Coast | None | 0.04 | 50 | P1_5F-50_ |
|  |  |  |  |  |  | 200 | P1_5F-200_ |
| 2 | M | 44 | Ivory Coast | NA | 0.08 | 50 | P2_5F-50_ |
|  |  |  |  |  |  | 200 | P2_5F-200_ |
| 3 | M | 45 | Cameroon | NA | 0.25 | 50 | P3_5F-50_ |
|  |  |  |  |  |  | 200 | P3_5F-200_ |
| 4 | M | 44 | Central African Republic | None | 0.4 | 50 | P4_5F-50_ |
|  |  |  |  |  |  | 200 | P4_5F-200_ |
| 5 | M | 29 | Ivory Coast | None | 5.5 | 50 | P5_5F-50_ |
|  |  |  |  |  |  | 200 | P5_5F-200_ |

Note – NA, Not Available. ^a^ % para, parasitemia in percentage.

**Table S2 – sWGA primers for *P. falciparum.***

| **Primer name** | **Primer sequence** |
| --- | --- |
| Pf1 | ATATATATAT*A |
| Pf2 | TATATATATAT*T |
| Pf3 | TATATATATA*A |
| Pf4 | TAATATATA*T |
| Pf5 | TATATATATT*T |
| Pf6 | ATTATTATTA*T |
| Pf7 | TAATAATAAT*A |
| Pf8 | AAAAAAAAAAA*A |
| Pf9 | AATAATAATA*A |
| Pf10 | TATTATATA*T |

* phosphorothioate bond.

**Table S3 – Content in *P. falciparum* species and total DNA before and after 5WBF measured by Qubit and qPCR DNA quantification.**

| **Patient** | **% para^a^** | **Blood volume (µL)** | **Sample name** | **Unfiltered/**  **5WBF** | **Qubit total DNA (ng/µL) ^b^** | ***H. sapiens* ΔCt ^c^** | ***P. falciparum* ΔCt ^c^** |
| --- | --- | --- | --- | --- | --- | --- | --- |
| 1 | 0.04 | 50 |  | Unfiltered | 2.07 | 16 | 1 |
|  |  |  | P1_5F-50_ | 5WBF | < 0.01 |  |  |
|  |  | 200 |  | Unfiltered | 12.1 | 14 | 1 |
|  |  |  | P1_5F-200_ | 5WBF | 0.05 |  |  |
| 2 | 0.08 | 50 |  | Unfiltered | 2.95 | 12 | 2 |
|  |  |  | P2_5F-50_ | 5WBF | 0.02 |  |  |
|  |  | 200 |  | Unfiltered | 20.4 | 14 | 1 |
|  |  |  | P2_5F-200_ | 5WBF | 0.09 |  |  |
| 3 | 0.25 | 50 |  | Unfiltered | 7.80 | 16 | 3 |
|  |  |  | P3_5F-50_ | 5WBF | 0.02 |  |  |
|  |  | 200 |  | Unfiltered | 19.30 | 15 | 1 |
|  |  |  | P3_5F-200_ | 5WBF | 0.30 |  |  |
| 4 | 0.40 | 50 |  | Unfiltered | 0.10 | >10 | 2 |
|  |  |  | P4_5F-50_ | 5WBF | < 0.01 |  |  |
|  |  | 200 |  | Unfiltered | 9.52 | 15 | 2 |
|  |  |  | P4_5F-200_ | 5WBF | 0.10 |  |  |
| 5 | 5.50 | 50 |  | Unfiltered | 2.39 | 15 | -1 |
|  |  |  | P5_5F-50_ | 5WBF | 0.22 |  |  |
|  |  | 200 |  | Unfiltered | 13.40 | 13 | -1 |
|  |  |  | P5_5F-200_ | 5WBF | 2.23 |  |  |

Note – ^a^ % para, parasitemia in percentage. ^b^ DNA concentration obtained from unfiltered or 5WBF-filtered blood was quantified using Qubit® dsDNA high sensitivity (Thermo Fisher Scientific); DNA elution volume was 200 µL for all samples. ^c^ Ct, cycle threshold; ΔCt = Ct_5WBF_ – Ct_unfiltered_.
